# Analysis of COVID-19 Risk Following a Ring Vaccination Intervention to Address SARS-CoV-2 Alpha Variant Transmission in Montreal, Canada

**DOI:** 10.1001/jamanetworkopen.2021.47042

**Published:** 2022-02-11

**Authors:** Simone Périnet, Geneviève Cadieux, Sarah-Amélie Mercure, Mylène Drouin, Robert Allard

**Affiliations:** 1 Direction régionale de santé publique de Montréal, Montreal, Quebec, Canada; 2 Canadian Field Epidemiology Program, Public Health Agency of Canada, Canada; 3 Department of Epidemiology, Biostatistics and Occupational Health, McGill University, Montreal, Quebec, Canada; 4 École de santé publique, Université de Montréal, Montreal, Quebec, Canada

## Abstract

**Importance:**

Given limited COVID-19 vaccine availability early in the pandemic, optimizing immunization strategies was of paramount importance. Ring vaccination has been used successfully to control transmission of other airborne respiratory viruses.

**Objective:**

To assess the association of a ring vaccination intervention on COVID-19 spread in the initial epicenter of SARS-CoV-2 Alpha variant transmission in Montreal, Canada.

**Design, Setting, and Participants:**

This cohort study compared COVID-19 daily disease risk in 3 population-based groups of neighborhoods in Montreal, Canada, defined by their intervention-specific vaccine coverage at the neighborhood level: the primary intervention group (500 or more vaccinated persons per 10 000 persons), secondary intervention group (95 to 499), and control group (0 to 50). The groups were compared within each of 3 time periods: before intervention (December 1, 2020, to March 16, 2021), during and immediately after intervention (March 17 to April 17, 2021), and 3 weeks after the intervention midpoint (April 18 to July 18, 2021). Data were analyzed between June 2021 and November 2021.

**Exposures:**

Vaccination targeted parents and teachers of children attending the 32 schools and 48 childcare centers in 2 adjacent neighborhoods with highest local transmission (case counts) of Alpha variant shortly after its introduction. Participants were invited to receive 1 dose of mRNA vaccine between March 22 and April 9, 2021 (before vaccine was available to these age groups).

**Main Outcomes and Measures:**

COVID-19 risk in 3 groups of neighborhoods based on intervention-specific vaccine coverage.

**Results:**

A total of 11 794 residents were immunized, with a mean (SD) age of 43 (8) years (range, 16-93 years); 5766 participants (48.9%) lived in a targeted neighborhood, and 9784 (83.0%) were parents. COVID-19 risk in the primary intervention group was significantly higher than in the control group before (unadjusted risk ratio [RR], 1.58; 95% CI 1.52-1.65) and during (RR, 1.63; 95% CI, 1.52-1.76) intervention, and reached a level similar to the other groups in the weeks following the intervention (RR, 1.03; 95% CI, 0.94-1.12). A similar trend was observed when restricting to SARS-CoV-2 variants and persons aged 30 to 59 years (before: RR, 1.72; 95% CI, 1.63-1.83 vs after: RR, 1.01; 95% CI, 0.88-1.17).

**Conclusions and Relevance:**

Our findings show that ring vaccination was associated with a reduction in COVID-19 risk in areas with high local transmission of Alpha variant shortly after its introduction. Ring vaccination may be considered as an adjunct to mass immunization to control transmission in specific areas, based on local epidemiology.

## Key Points

**Question:**

Is ring vaccination targeting contacts of confirmed cases and persons who are in close contact with these contacts useful for controlling local SARS-CoV-2 transmission following the introduction of a new variant?

**Findings:**

In this cohort study of 106 Montreal neighborhoods, ring vaccination was associated with a reduction in COVID-19 incidence in areas with high SARS-CoV-2 Alpha variant case counts.

**Meaning:**

These results suggest that ring vaccination may be considered an adjunct to mass immunization to control transmission in specific areas where new variants are first introduced, based on local epidemiology.

## Teaser

This cohort study examines risk of COVID-19 infection following the implementation of ring vaccination measures in high-incidence neighborhoods in Montreal, Canada, during the Alpha variant outbreak.

## Introduction

Early randomized controlled trials of vaccines against SARS-CoV-2 were designed to assess efficacy against COVID-19 disease, hospitalization, and death^1,2,3,4^; evidence regarding a reduced risk of asymptomatic infection emerged later.^5,6,7^ Available evidence and limited vaccine supply led many countries to adopt a vaccine prioritization scheme similar to influenza, which aims to reduce severe morbidity and mortality, and mitigate the burden of COVID-19 spread on health care systems^8,9,10,11,12,13,14,15^ rather than to control transmission. This prioritization scheme led some jurisdictions, like the province of Quebec in Canada, to allocate vaccine doses based primarily on the size of the populations prioritized for vaccination in each administrative region, and to not formally take into account the number of COVID-19 cases in each region. This prioritization scheme missed the opportunity to use immunization as a means to control transmission in areas with high COVID-19 case counts.

Indeed, there is evidence from other communicable diseases that immunization can be a tool not only to prevent disease but also to stop transmission. Ring vaccination, as opposed to mass vaccination, targets contacts of confirmed cases, as well as persons who are in close contact with these contacts, with the ultimate aim of stopping transmission. It was first developed against smallpox^16^ and has been used successfully to contain measles outbreaks in underimmunized communities.^17,18,19^ Its potential effectiveness against Ebola virus disease was suggested by epidemic modeling^20,21^ and confirmed by a randomized controlled trial comparing immediate with delayed vaccination.^22^ Modeling also supports the effectiveness of ring vaccination against COVID-19,^23^ and it has been recommended as a supplementary strategy against this infection in medium- or high-risk areas within low-incidence countries.^24^

With mounting evidence of vaccine effectiveness against COVID-19 and asymptomatic SARS-CoV-2 infection,^5,6,7^ as well as early evidence of efficacy against the Alpha variant,^25,26,27,28,29^ when a surge in COVID-19 transmission among emergency shelter users and staff was detected in December 2020, Montreal Public Health, with the collaboration of its health care system and community partners, undertook a ring vaccination intervention of contacts and the contacts of contacts of cases among its shelter users and staff in January and February 2021. Whereas this intervention seemed to contribute to rapidly bringing COVID-19 transmission under control in the target population, the opportunity to evaluate ring vaccination and its potential limitations was not available because of the absence of a control group. Based on this experience, Montreal Public Health then sought to assess COVID-19 risk following a ring vaccination intervention around childcare center and school attendees, which have been observed to be important drivers of transmission, in the initial epicenter of SARS-CoV-2 Alpha variant transmission in Montreal in comparison with other areas neither targeted nor reached by the intervention.

## Methods

### Study Design and Participants

The objective of the ring vaccination intervention was to suppress transmission of the Alpha variant in Montreal by targeting its initial epicenter. The neighborhoods in Montreal where sustained local transmission of Alpha variant with high case counts was first detected experienced multiple introductions of Alpha variant related to travel to and from New York State, where Alpha variant transmission was higher. These neighborhoods also had large households with multiple children who attended childcare centers and schools, and had multiple contacts related to participation in social and religious events.

The proof-of-concept ring vaccination intervention was initially designed to offer, over a 2-week period, 1 dose of a COVID-19 vaccine to all adults residing in the 2 neighborhoods with the highest number of new cases of presumptive SARS-CoV-2 Alpha variant in the previous 2 weeks, with the aim of reaching 70% first-dose immunization coverage. The intervention was conceived in early March 2021, when only older persons and health care workers were eligible for immunization in the province of Quebec, and immunization coverage targets (first dose) of 75% had just been reached in those populations.

Because of limited vaccine supply and provincial government restrictions, the size of the population targeted by the intervention had to be reduced. At the time, over a third of all new COVID-19 cases in the targeted neighborhoods were aged less than 18 years, and in-person childcare center and school attendance were observed to be a major driver of overall transmission, in addition to transmission within households. However, none of the vaccines were yet authorized for use in children. Based on the patterns of transmission observed, it was decided to target exclusively parents of children attending childcare centers or schools in an area comprised of 2 adjacent neighborhoods with the highest presumptive Alpha variant case count in Montreal. In response to concerns raised by personnel, schoolteachers and childcare center educators were later added to the target population although they were not thought to be major drivers of transmission. Given the high case counts in these neighborhoods, many parents and teachers would have been considered contacts or contacts of contacts of cases of Alpha variant.

A total of 32 schools and 48 childcare centers were identified in targeted neighborhoods. Parents were identified based on lists of childcare center and school attendees; teachers and educators were identified by childcare center and school administrators. Letters were sent to parents on March 19, 2021, and to teachers and educators on March 21, describing the intervention and inviting them to schedule an appointment to receive 1 dose of an mRNA vaccine at 1 of 4 nearby mass immunization sites, between March 22 and April 9, 2021. Appointments were available through the provincial online appointment scheduler or by telephone. Three of these sites were already in operation as part of the immunization campaign under way in Montreal, an additional site was opened; hours of operation included evenings and weekends. At each vaccination site, participant eligibility was confirmed through presentation of the letter invitation, proof of child enrollment in one of the targeted childcare centers or schools, and valid identification. The intervention was also promoted through local media.

We used a quasi-experimental design with a nonrandomized untreated control group, dependent pretest and posttest samples, and a double pretest^30^ to evaluate this ring vaccination intervention. The compared groups were defined post hoc using Montreal neighborhoods, ie, 111 geographic areas that aim to depict a more accurate representation of the city’s local communities than other administrative geographical units.^31^ Ethical review of public health program evaluations is not required in Canada as per article 2.5 of the federal government’s interagency advisory panel on research ethics.^32^

One hundred and six neighborhoods (95% of Montreal neighborhoods, representing a total of 1 843 290 residents) were retrospectively assigned to 1 of 3 groups according to the proportion of their population vaccinated as part of the ring vaccination intervention. The primary intervention group comprised 4 adjacent neighborhoods: 2 with the targeted childcare centers and schools and 2 adjacent neighborhoods where many families and staff resided. All had an intervention-specific immunization rate of 500 or more per 10 000 persons. The secondary intervention group comprised 11 neighborhoods surrounding the primary intervention group, with an intervention-specific immunization rate between 95 and 499 per 10 000 persons. The control group represented neighborhoods that had very low (0 to 50 per 10 000 persons) intervention-specific vaccine coverage and included many neighborhoods distributed across the city. Five neighborhoods were excluded from the evaluation as they achieved moderate vaccine coverage as part of the intervention (between 51 and 94 per 10 000).

The 2 intervention groups were compared with a nonrandomized control group within each of 3 time periods: (1) before the ring vaccination intervention (December 1, 2020, to March 16, 2021), (2) in a stabilization period overlapping with the intervention period and before a potential effect could be observed because of the delay between vaccine administration, development of immunity, and eventual case reporting (March 17 to April 17, 2021), and (3) 3 weeks after the immunization efforts’ midpoint until the end of the Quebec-wide third wave of the pandemic (April 18 to July 18, 2021). The 3-week delay after the midpoint was determined a priori and was informed by dose distribution data (not shown).

### Data Sources

An intervention-specific extraction of the provincial immunization registry was provided by the health care team in charge of logistical aspects. These data were used for descriptive analysis of the population reached by the intervention. Data on COVID-19 cases were extracted from the provincial COVID-19 surveillance database on September 1, 2021; a variable entered at the time of case investigation indicated whether cases were confirmed (ie, by whole genome sequencing) or presumptive (ie, by variant-specific reverse transcription–polymerase chain reaction [RT-PCR] or S-gene target failure) variants of concern. Population-level data on COVID-19 cases were used for the evaluation. Data on SARS-CoV-2 tests were extracted from the provincial COVID-19 testing results database on May 28, 2021. Data on immunizations against COVID-19 were extracted from the Quebec immunization registry (Système d’information pour la protection en maladies infectieuses [SI-PMI]). 2016 Canadian census data were used for all denominators as well as sociodemographic indicators at the neighborhood level. Racial and ethnic data classification followed the Statistics Canada designation of visible minority, which includes individuals identifying as South Asian, Chinese, Black, Filipino, Arab, Latin American, Southeast Asian, West Asian, Korean, and Japanese.^33^

### Statistical Analysis

We performed a descriptive time-series analysis of disease risk smoothed using a 7-day moving average. We compared risk across groups using unadjusted risk ratios (RR) and their 95% confidence intervals for all cases and for cases presumptively infected with a SARS-CoV-2 variant of concern (mostly Alpha) to confirm whether the observations were statistically significant at a *P* < .05 significance level. The primary intervention group and the secondary intervention group were each compared with the control group, within 3 time periods. COVID-19 risk and risk ratios (RR) were calculated for all age groups, as well as for persons aged 30 to 59 years, the age group most reached by the intervention. Data analyses were performed using the SAS base software version 9.4 (SAS Institute) and Microsoft Excel 2016.

## Results

A total of 13 488 persons, including 11 794 Montreal residents (95.3%; of 12 379 persons with area of residence data), were immunized with 1 dose of an mRNA vaccine between March 22 and April 9, 2021, as part of the ring vaccination intervention. Immunized residents ranged from ages 16 to 93 years, the mean (SD) age was 43 (8) years. Most (92.3%) were aged 30 to 59 years and 11 047 (81.9%) were parents. Approximately half of Montreal residents immunized as part of the intervention resided in the 4 adjacent neighborhoods that comprised the primary intervention group (5766 [48.9%]); 3892 (33%) parents, teachers and educators reached by the intervention lived in surrounding neighborhoods (Table 1). Estimated vaccine uptake among eligible individuals was very high, ranging from 94% in parents to 98% in all eligible persons (data not shown).

**Table 1.** Characteristics of the Individuals Immunized as Part of the Ring Vaccination Intervention

Characteristic	Individuals, No. (%)			
	Residents of Montreal (n = 11 794)	Residents of other regions (n = 585)	Region of residence not available (n = 1109)	Total (n = 13 488)
Age, mean (SD) [range], y	43 (8) [16-93]	44 (8) [21-69]	41 (10) [20-76]	43 (8) [16-93]
Age group				
16-19 y	39 (0.3)	0	0	39 (0.3)
20-24 y	213 (1.8)	14 (2.4)	18 (1.6)	245 (1.8)
25-29 y	392 (3.3)	30 (5.1)	41 (3.7)	463 (3.4)
30-34 y	944 (8.0)	54 (9.2)	143 (12.9)	1141 (8.5)
35-39 y	2149 (18.2)	86 (14.7)	273 (24.6)	2508 (18.6)
40-44 y	2865 (24.3)	112 (19.2)	301 (27.1)	3278 (24.3)
45-49 y	2600 (22.1)	109 (18.6)	171 (15.4)	2880 (21.4)
50-54 y	1604 (13.6)	91 (15.6)	101 (9.1)	1796 (13.3)
55-59 y	740 (6.3)	61 (10.4)	40 (3.6)	841 (6.2)
≥60 y	248 (2.1)	28 (4.8)	21 (1.9)	297 (2.2)
Eligibility group				
Parent	9784 (83.0)	298 (50.9)	965 (87.0)	11 047 (81.9)
Teacher	831 (7.1)	165 (28.2)	44 (4.0)	1040 (7.7)
Daycare educator	433 (3.7)	68 (11.6)	30 (2.7)	531 (3.9)
Other daycare or school employee	247 (2.1)	42 (7.2)	24 (2.2)	309 (2.3)
Data not available	46 (4.2)	499 (2.1)	46 (4.1)	557 (4.1)
Geographical area of residence				
Primary intervention group neighborhood	5766 (48.9)	NA	NA	5766 (42.8)
Secondary intervention group neighborhood	3895 (33.0)	NA	NA	3895 (28.9)
Control group neighborhood	1354 (11.5)	NA	NA	1354 (10.0)
Other neighborhood (excluded)	721 (6.1)	NA	NA	721 (5.4)
Neighborhood not available or not applicable	58 (0.5)	585 (100)	1109 (100)	1752 (13.0)

Table 2 summarizes the baseline characteristics of the population in the 3 groups of neighborhoods used to evaluate the ring vaccination intervention. Compared with the control group, the primary intervention group included more individuals aged 0 to 24 years (27 460 of 86 950 [31.6%] vs 421 640 of 1 546 625 [27.3%]) and fewer adults aged 25 to 59 years (39 360 [45.3%] vs 779 180 [50.4%]). A smaller proportion of participants had no certificate, diploma, or degree (13.4%; 95% CI, 13.2%-13.7% vs 18.4%; 95% CI, 18.3%-18.5%); a smaller proportion had low income according to census guidelines (15.0%; 95% CI, 8.1%-21.8% vs 18.2%; 95% CI, 16.4%-20.0%); and a larger proportion self-identified as a visible minority (45.8%; 95% CI, 45.4%-46.1% vs 32.5%; 95% CI, 32.4%-32.5%). Based on previous COVID-19 infections reported to public health and immunization records, a slightly smaller proportion (83.4%; 95% CI, 83.1%-83.6%) of the primary intervention group was susceptible to SARS-CoV-2 at baseline compared with the secondary intervention and control groups (87.6%; 95% CI, 87.5%-87.6%), which is consistent with a higher 6-month cumulative COVID-19 risk in this group.

**Table 2.** Comparison of Baseline Characteristics of the Population Residing in the 3 Intervention Groups

Characteristic	Primary intervention group		Secondary intervention group		Control group	
	No.	% (95% CI)	No.	% (95% CI)	No.	% (95% CI)
Composition of groups						
No. of Montreal neighborhoods	4	NA	11	NA	91	NA
Total population	86 950	NA	209 715	NA	1 546 625	NA
Age groups						
0-14 y	15 505	17.8 (17.6-18.1)	35 195	16.8 (16.6-16.9)	237 565	15.4 (15.3-15.4)
15-24 y	11 955	13.8 (13.5-14.0)	29 295	14.0 (13.8-14.1)	184 075	11.9 (11.9-12.0)
25-59 y	39 360	45.3 (44.9-45.6)	97 145	46.3 (46.1-46.5)	779 180	50.4 (50.3-50.5)
60 and over	20 145	23.2 (22.9-23.5)	48 075	22.9 (22.7-23.1)	345 840	22.4 (22.3-22.4)
Other demographic information						
Population without a certificate, diploma, or degree^a^	9390	13.4 (13.2-13.7)	16 795	9.9 (9.7-10.0)	233 530	18.4 (18.3-18.5)
Low income, mean^b^	NA	15.0 (8.1-21.8)	NA	15.8 (11.9-19.7)	NA	18.2 (16.4-20.0)
Self-identified as visible minority^c^	39 070	45.8 (45.4-46.1)	58 535	28.5 (28.3-28.7)	489 300	32.5 (32.4-32.5)
COVID-19 epidemiological information						
Cases and cumulative risk (per 100 000), No.^d^	5619	6462 (6300-6630)	6967	3322 (3240-3400)	63 534	4107 (4080-4140)
Intervention-specific vaccine coverage (per 10 000), No.^e^	5766	663 (647-680)	3895	186 (180-192)	1354	9 (9-9)
Total vaccine coverage (per 10 000), No.^e^^,^^f^	8820	1014 (994-1034)	20 004	954 (942-967)	128 900	833 (829-837)
Susceptible population^g^	72 511	83.4 (83.1-83.6)	182 744	87.1 (87.0-87.3)	1 354 191	87.6 (87.5-87.6)

^a^ Census question denominators were 69 950 individuals in the primary intervention group, 169 945 in the secondary intervention group, and 1 270 660 in the control group.

^b^ Data available as the percentage of population self-reporting low income based on Statistics Canada’s low-income cut-offs after taxes.

^c^ Racial and ethnic data classification follows the Statistics Canada designation of visible minority, which includes individuals identifying as South Asian, Chinese, Black, Filipino, Arab, Latin American, Southeast Asian, West Asian, Korean, and Japanese.^33^Census question denominators were 85 355 individuals in the primary intervention group, 205 150 in the secondary intervention group, and 1 507 795 control group.

^d^ Cumulative COVID-19 cases reported in the 6-month period prior to the beginning of the intervention (between September 21, 2020, and March 21, 2021), based on neighborhood of residence.

^e^ Vaccine coverage includes individuals receiving at least 1 dose.

^f^ Proportion of the population who received their first vaccine dose on or before March 7, 2021 (14 days from March 22).

^g^ Susceptible population includes individuals who were COVID-19 naive (ie, no infection 6 months before the intervention) and who received zero doses of COVID-19 vaccine, calculated based on the number of cases and the number of persons who received at least 1 vaccine dose. Because this estimate assumes no overlap between COVID-19 cases and vaccinated persons, it likely underestimates the true proportion of susceptible individuals.

At baseline, COVID-19 daily risks were substantially higher in the primary intervention group compared with the control group and secondary intervention group (Figure). The primary intervention group had an approximately 60% greater risk of COVID-19 and a 9 times greater risk of infection with a variant of concern compared with the control group (RR, 9.43; 95% CI, 8.43-10.55) (Table 3). During the intervention period, the COVID-19 daily risk in the primary intervention group remained stable and statistically significantly higher than the control group and secondary intervention group (RR, 1.63; 95% CI, 1.52-1.76) (Figure and Table 3). Over the course of the postintervention period, disease risk showed a steep decline in the primary intervention group only, and the observed differences in disease risk between groups were no longer statistically significant (RR, 1.03; 95% CI, 0.94-1.12) (Figure and Table 3). A similar temporal pattern was observed for variant of concern–specific risk (intervention: RR, 1.89; 95% CI, 1.69-2.11 vs postintervention: RR, 1.15; 95% CI, 1.04-1.26). A smaller decrease in disease risk was observed for the secondary intervention group for variant of concern cases that was statistically significant (intervention: RR, 1.27; 95% CI 1.17-1.39 vs postintervention: RR, 0.88; 95% CI, 0.82-0.94) (Table 3). The RRs before and during the intervention in the 30- to 59-year-old age group were in general slightly higher than for the overall sample (eg, intervention period: RR, 1.79; 95% CI, 1.60-2.00), demonstrating a larger effect size in this age group. An age-stratified analysis (data not shown) demonstrated similar effects measures in 10-year age strata within the 30 to 59 age group, suggesting no confounding by age. The observed trends were maintained throughout the Quebec-wide third wave (March 21 to July 17, 2021) amidst a small increase in incidence in May.

**Figure.**
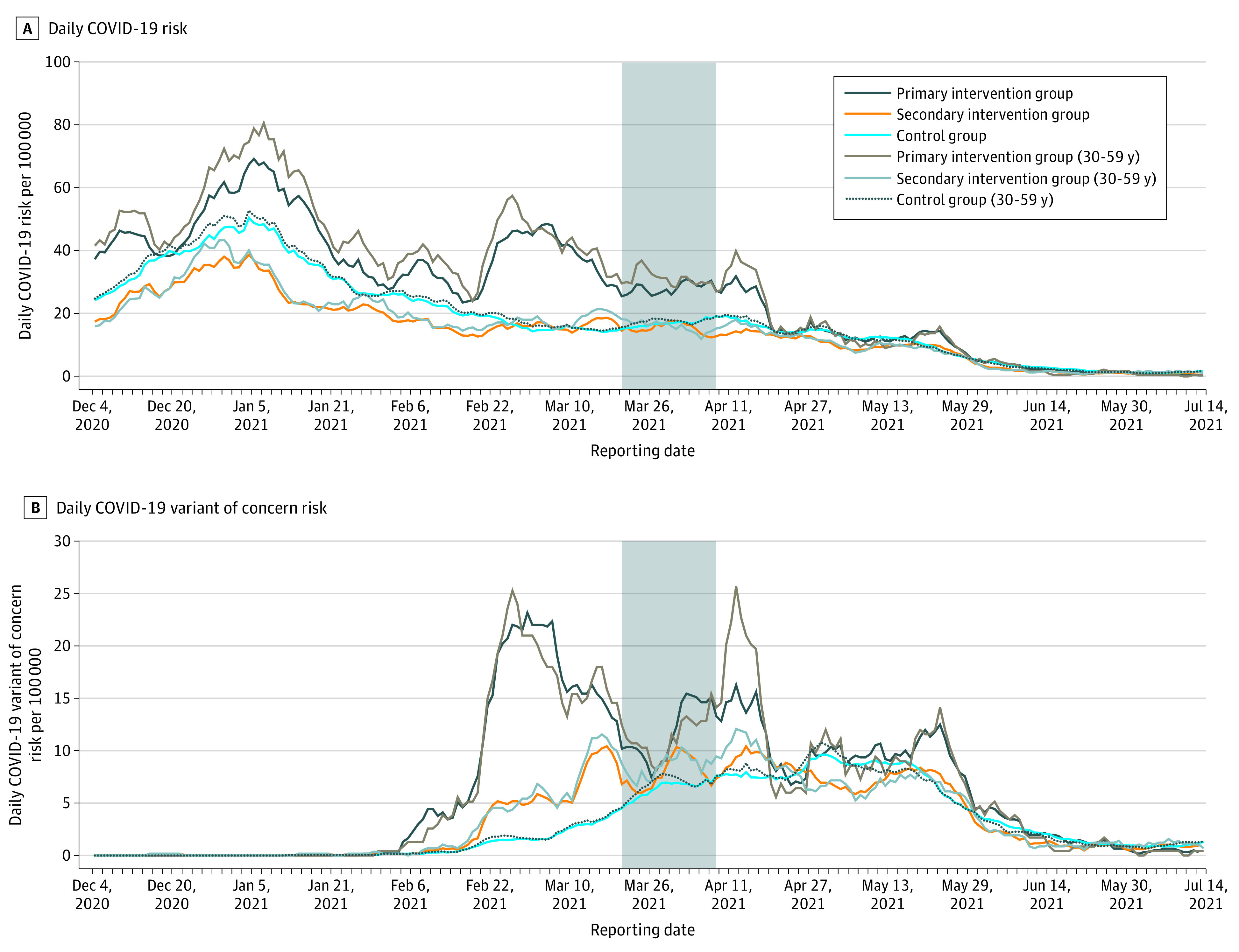
Time Series of COVID-19 and COVID-19 Variant of Concern Risk per 100 000 Population The highlighted area indicates the ring vaccination intervention period. The variant of concern over the intervention period was almost exclusively the Alpha variant (2974 of 3087 [96.3%] variant of concern cases between December 2020 and April 2021).

**Table 3.** Comparison of Unadjusted Risk Ratios for Infection With Any SARS-CoV-2 and Variants of Concern

Variable	RR (95% CI)^a^		
	Preintervention^b^	Intervention^c^	Postintervention^d^
All cases			
Control group	1 [Reference]	1 [Reference]	1 [Reference]
Primary intervention group	1.58 (1.52-1.65)	1.63 (1.52-1.76)	1.03 (0.94-1.12)
Secondary intervention group	0.76 (0.74-0.79)	0.89 (0.83-0.95)	0.81 (0.76-0.86)
Variant of concern cases^e^			
Control group	1 [Reference]	1 [Reference]	1 [Reference]
Primary intervention group	9.43 (8.43-10.55)	1.89 (1.69-2.11)	1.15 (1.04-1.26)
Secondary intervention group	2.88 (2.54-3.27)	1.27 (1.17-1.39)	0.88 (0.82-0.94)
All cases aged 30-59 y			
Control group	1 [Reference]	1 [Reference]	1 [Reference]
Primary intervention group	1.72 (1.63-1.83)	1.79 (1.60-2.00)	1.01 (0.88-1.17)
Secondary intervention group	0.79 (0.74-0.83)	0.93 (0.84-1.03)	0.83 (0.75-0.92)
Variant of concern cases aged 30-59 y^e^			
Control group	1 [Reference]	1 [Reference]	1 [Reference]
Primary intervention group	8.16 (6.83-9.76)	2.04 (1.72-2.41)	1.10 (0.93-1.30)
Secondary intervention group	2.78 (2.30-3.37)	1.36 (1.19-1.55)	0.87 (0.77-0.98)

Abbreviation: RR, risk ratio.

^a^ Significance for RR indicated by all results with 95% CI ranges that do not cross 1.00.

^b^ The preintervention period includes January 1, 2021, to March 16, 2021.

^c^ The ring vaccination intervention period included time before a potential intervention effect could be expected, from March 17, 2021, to April 17, 2021.

^d^ The postintervention period ranged from the intervention midpoint until the end of the Quebec-wide third wave of the pandemic, from April 18, 2021, to July 18, 2021.

^e^ Variant of concern cases in the intervention period were almost exclusively Alpha variant cases (2974 of 3087 [96.3%] variant of concern cases between December 2020 and April 2021).

Of note, a mass immunization campaign that was underway concurrently in Montreal. The proportion of persons vaccinated as part of the mass immunization campaign was similar in our primary (21.7%; 95% CI, 21.4%-21.9%) and secondary (22.3%; 95% CI, 22.1%-22.5%) intervention groups compared with the control group (22.0%, 95% CI 21.9-22.0) as of the end date of the intervention.

## Discussion

Using a controlled quasi-experimental design, this cohort study found a statistically significant association between ring vaccination with 1 dose of an mRNA vaccine and a reduction in COVID-19 risk in Montreal. Neighborhoods reached by ring vaccination experienced a steep decrease in COVID-19 risk 2 to 3 weeks after the intervention compared with neighborhoods not reached by ring vaccination. These trends and associations are also observed among individuals aged 30 to 59 years, who were the most targeted and reached by ring vaccination. The timing of the observed reduction in disease risk is consistent with the delay required to achieve immunity after administration of the first dose of an mRNA vaccine. These results are compatible with a population-level effect of this ring vaccination intervention. In fact, this intervention, deployed within just a few weeks of the first detection of the Alpha variant in Montreal, is thought to have significantly contributed to the flattening of the third wave and to the slowing of the replacement of the ancestral SARS-CoV-2 strain with the Alpha variant.

To our knowledge, this is the first real-world study of a ring vaccination intervention for COVID-19. A modeling study^23^ showed that with a limited number of doses of vaccine, if postexposure prophylaxis is at all effective, the most efficient way to control COVID-19 is ring vaccination; however, at least 90% of contacts per case needs to be traced and vaccinated. Studies have demonstrated the effectiveness of ring vaccination for controlling other aerosol-transmitted infections, such as smallpox^16^ and measles.^17,19^

### Limitations

The main limitation of our study stems from the nonrandom allocation of the intervention; as a result, other factors may have contributed to the decreased incidence observed in the primary intervention group. One such factor was the mass immunization campaign that was underway concurrently in Montreal; however, data suggests that the proportion of persons vaccinated as part of the mass immunization campaign was similar in our primary (21.7%) and secondary (22.3%) intervention groups compared with the control group (22.0%) as of the end date of the intervention.

Differential exhaustion of susceptible individuals between the intervention and control groups could in theory have accounted for the observed effect. However, given that over 80% of the population in the primary intervention group was considered susceptible to COVID-19 at baseline, it is unlikely that the exhaustion of susceptible people in the primary intervention group played a role. Furthermore, if exhaustion of the susceptible population had occurred in the primary intervention group, the latter’s COVID-19 incidence rate should have decreased to less than that of the control group, but this was not observed.

Another potential factor that may have led to a differential decrease in disease risk after the intervention is reduced participation in COVID-19 testing in the intervention groups compared with the control group; however, daily testing rates remained similar across the 3 groups. Before the intervention, the rate was 2 persons tested daily per 1000 persons in all 3 groups, and 3 weeks after the intervention midpoint, the daily testing rates were 14 and 13 individuals per 1000 for the primary and secondary intervention groups, respectively, and 14 per 1000 for the control group. Unadjusted measures of association are presented; however, given that socioeconomic level, age, and population susceptibility to COVID-19 must have remained largely unchanged in the short evaluation period, it is unlikely that these variables confounded our results.

Before the intervention, the risk ratio for all COVID cases (as opposed to Alpha variant cases) in the secondary intervention group was lower than in the control group; this trend was observed as far back as 6 months prior to the start of the intervention (and possibly earlier) and may have been partially related to differences in socioeconomic or other factors. However, when looking at Alpha variant cases, the risk in the primary and secondary intervention groups were both higher than in the control group, which is consistent with the epidemiological situation at the time (ie, sustained local transmission of Alpha variant first occurring in those neighborhoods).

Whereas behavior change other than vaccination was not targeted by our ring vaccination intervention, the intervention groups may have modified other behaviors that affect COVID-19 risk (eg, mask-wearing, physical distancing) as a result of the intervention, which may have contributed to the reduction in disease risk observed postintervention. Therefore, the observed effect should be considered as associated with the overall ring vaccination intervention rather than the vaccine alone.

Finally, our analyses, including our investigations of potential confounding, were performed at the population or group level after aggregating neighborhood-level data, including those on prior COVID-19 infections. Residual confounding at the individual level was possible. A randomized controlled trial, if feasible, would be needed to confirm our findings.

## Conclusions

Evidence from this study supports the conclusion that strategies aimed at controlling transmission are associated with long-term reduction in morbidity, mortality, and other impacts of the pandemic and the preservation of health care system capacity.^32,34,35,36,37^ Our findings suggest that planners of COVID-19 immunization strategies should strongly consider adding ring vaccination as an adjunct to mass immunization in order to control transmission in specific areas based on local epidemiology. More generally, after persons at highest risk of hospitalization and death are immunized, consideration should be given to targeting immunization to persons residing, working, or attending school in areas with high or growing COVID-19 case counts, with the goal of stopping or slowing transmission.
